# Google Street View Derived Built Environment Indicators and Associations with State-Level Obesity, Physical Activity, and Chronic Disease Mortality in the United States

**DOI:** 10.3390/ijerph17103659

**Published:** 2020-05-22

**Authors:** Lynn Phan, Weijun Yu, Jessica M. Keralis, Krishay Mukhija, Pallavi Dwivedi, Kimberly D. Brunisholz, Mehran Javanmardi, Tolga Tasdizen, Quynh C. Nguyen

**Affiliations:** 1 Department of Public Health Science, University of Maryland School of Public Health, College Park, MA 20742, USA; 2 Department of Epidemiology and Biostatistics, University of Maryland School of Public Health, College Park, MD 20742, USA; wyu1227@umd.edu (W.Y.); jkeralis@umd.edu (J.M.K.); pdwived1@umd.edu (P.D.); 3 The Harker School, San Jose, CA 95129, USA; 21krishaym@students.harker.org; 4 Intermountain Healthcare Delivery Institute, Intermountain Healthcare, Murray, UT 4107, USA; kim.brunisholz@imail.org; 5 Department of Electrical and Computer Engineering, Scientific Computing and Imaging Institute, University of Utah, Salt Lake City, UT 84112, USA; mehjavan@gmail.com (M.J.); tolga@sci.utah.edu (T.T.)

**Keywords:** google street view, built environment, obesity, physical activity, diabetes, cardiovascular disease, mortality, big data

## Abstract

Previous studies have demonstrated that there is a high possibility that the presence of certain built environment characteristics can influence health outcomes, especially those related to obesity and physical activity. We examined the associations between select neighborhood built environment indicators (crosswalks, non-single family home buildings, single-lane roads, and visible wires), and health outcomes, including obesity, diabetes, cardiovascular disease, and premature mortality, at the state level. We utilized 31,247,167 images collected from Google Street View to create indicators for neighborhood built environment characteristics using deep learning techniques. Adjusted linear regression models were used to estimate the associations between aggregated built environment indicators and state-level health outcomes. Our results indicated that the presence of a crosswalk was associated with reductions in obesity and premature mortality. Visible wires were associated with increased obesity, decreased physical activity, and increases in premature mortality, diabetes mortality, and cardiovascular mortality (however, these results were not significant). Non-single family homes were associated with decreased diabetes and premature mortality, as well as increased physical activity and park and recreational access. Single-lane roads were associated with increased obesity and decreased park access. The findings of our study demonstrated that built environment features may be associated with a variety of adverse health outcomes.

## 1. Introduction

The built environment is a structural determinant of public health, impacting health outcomes through its effects on physical activity patterns [1] and psychological well-being [2]. Previous studies have demonstrated associations between the built environment and long-term health outcomes and related behaviors, including body mass index (BMI) [3], physical activity [4], and mortality [5]. Being physically active lowers individuals’ risk of being overweight or obese [6] and is linked to a variety of other long-term health outcomes [7].

Evidence of the ability of the built environment to drive long-term health outcomes can be seen in the health disparities between populations living in historically segregated neighborhoods. Discriminatory policies such as redlining, a real estate practice discriminating against money or credit borrowers from certain areas with poverty in the United States, has resulted in uneven urban development, creating distinct geographic areas that still face systemic disadvantages [8]. Many of these neighborhoods have built environment features that are detrimental to residents’ long-term health, such as more alcohol outlets [9] and fast food restaurants, fewer recreational facilities [10], and higher levels of intra-urban heat [11].

Google Street View (GSV) (Google LLC, Mountain View, CA, United States) is a massive repository of digital images of streets and intersections around the world [12]. While in the past, information on physical neighborhood features had to be manually collected and characterized [13] or gleaned from administrative records [14], GSV images—which can be downloaded in bulk via an application programming interface (API)—can be used to identify features of the built environment relevant to public health across large geographic regions. By harnessing technological advancement in deep learning, GSV images can be used to identify neighborhood features with high levels of accuracy and reliability [15,16,17]. Google Street View is an underutilized big data source that allows for increased efficiency in data collection and analyses of built environment features as they relate to health.

In this study, we measured associations between built environment features and health outcomes. Specifically, we created indicators for crosswalks, non-single family home buildings, single-lane roads, and visible wires. The crosswalks indicator was chosen to describe the walkability of an area, which, when increased, has been shown by previous studies to be associated with decreases in negative health outcomes. For example, a study found that a higher level of neighborhood walkability was related to decreases in unfavorable health outcomes like overweight status, obesity, and diabetes [18]. Non-single family home buildings were chosen as an indicator of mixed land use/urbanicity. Previous analyses we have conducted measuring urban development have indicated that increased urban development is associated with decreases in chronic health morbidities and physical inactivity [19,20]. The single-lane roads indicator was selected because single-lane roads are more common in suburban and rural areas than urban areas. More single-lane roads indicate less urban development. Previous studies have found that rural areas have higher adverse health outcomes, like increased obesity [21] and cardiovascular disease [22], as well as decreased likelihood of physical activity [23]. Lastly, the visible wires indicator was chosen as an indicator of physical disorder, which has been shown in previous studies to be associated with chronic health conditions and mortality [19]. Few studies currently exist using this indicator to evaluate the built environment, such as a study in Rio de Janeiro that used the presence of visible wires to represent unsightly scenery and risk of electrical fires [24]. Through the selection of this indicator, we aim to add to the amount of available literature about visible wires as an environmental indicator for future research.

We used GSV images to create these built environment indicators, aggregating them at the state level across the United States, to predict associations with health outcomes such as physical activity, obesity or overweight status, and diabetes or cardiovascular disease mortality. We hypothesize that an increase in crosswalks and non-single family home buildings will be associated with an increase in physical activity and a decrease in chronic conditions and mortality, while an increase in single-lane roads and visible wires will be associated with a decrease in physical activity and an increase in chronic conditions and mortality. In doing so, we hope to further contribute to the growing literature regarding the use of Google Street View-derived built environment indicators to assess determinants of health outcomes, especially for indicators that have yet to be studied in depth.

## 2. Materials and Methods

Using national road network data, we built a database of latitude and longitude coordinates representing all the street intersections in the United States. We focused on sampling images from street intersections in order to create a dataset that characterizes environments where people inhabit. In the United States, there are vast, sparsely populated roadless areas, especially mountain ranges and deserts. The roadway network files were accessed from the 2017 Census Topologically Integrated Geographic Encoding and Referencing data set [25]. We downloaded all road types. We identified street intersections using PostgreSQL [26] (an open-source object-relational database system) with the PostGIS plugin, a spatial database extender that enables location queries to be run in structured query language (SQL).

We retrieved GSV images for street intersections using the corresponding coordinates identified from PostgreSQL. Between 15 December 2017 and 17 July 2018, we used Google’s Street View Image application programming interface (API) to obtain images. In total, we collected over 31 million images (31,247,167) from across the United States. Parameters for the API included the following: image size (640 × 640 pixels is the maximum image resolution for non-premium plan users), geographic location (geographic coordinates or addresses), field of view (zoom level), up or down angle of the camera relative to the Street View vehicle (default is 0), and heading (direction the camera is facing with 0 = north, 90 = east, 180 = south, and 270 = west). We obtained four Street View images (directions: west, east, north, and south) for each pair of coordinates to comprehensively capture 360-degree views of the environment. Image resolution was 640 × 640 pixels.

Convolutional Neural Networks (ConvNets) [27] achieve state-of-the-art accuracy for several computer vision tasks including object recognition, object detection, and scene labeling [28]. The neighborhood characteristics are: (1) presence of a crosswalk (yes/no), (2) presence of non-single-family home (single-family detached house vs. other), (3) single lane road (road without dividing line, yes/no), and (4) visible utility wires overhead (yes/no). Images were manually labeled by four of the authors. We manually annotated 18,700 images (from Chicago, Illinois, Charleston, West Virginia, and a random subset of images from across the United States) for these neighborhood characteristics. Inter-rater agreement was above 85% for all neighborhood indicators. We randomly divided each labeled image dataset into a training set (80%), used to calibrate the model; and a test set (20%), used to evaluate the trained model’s accuracy. We used a deep convolutional network (Visual Geometry Group (VGG-16 model) [29] that is commonly used for object recognition. We trained separate networks for each neighborhood indicator and achieved high accuracies (85%–93%) for the separate recognition tasks.

We obtained chronic disease outcomes data from the Centers for Disease Control and Prevention (CDC) Chronic Disease Indicators (CDI) database [30]. For physical activity, obesity, and overweight outcomes data collection, data was collected from CDC’s Division of Nutrition, Physical Activity, and Obesity (DNPAO) data, trend, and maps [31].

The CDC collects health outcome data on the state level, categorizing the data with Federal Information Processing (FIPS) two-digit codes that each identify a United States state or territory. We retrieved 12 outcome indicators: (1) obesity and (2) overweight status of adults (percentage of adults aged 18 years or older with an obesity and overweight classification, respectively, in 2017), (3) obesity and (4) overweight status of adolescents (percentage of teenagers in grades 9–12 with an obesity and overweight classification, respectively, in 2017), (5) diabetes in adults (prevalence of adults aged 18 years or older with diagnosed diabetes in 2016), (6) aerobic physical activity in adults (percentage of adults who had at least 150 minutes of moderately intense aerobic physical activity, or 75 minutes of vigorously intense aerobic activity, or an equivalent combination per week in 2017), (7) daily physical activity in adolescents (percentage of teenagers in grades 9–12 who had one hour or more of moderate to vigorous physical activity on a daily basis in 2017), (8) premature mortality (cases of premature mortality per 100,000 among adults aged 45 to 64 years), (9) diabetes mortality (cases of diabetes-related mortality per 100,000 in 2014), (10) cardiovascular disease mortality (cases of cardiovascular disease-related mortality per 100,000 in 2014), (11) park access (percent of USA population living within 1/2 mile of a park in 2015), and (12) youth recreational access (percent of youth with playgrounds, community centers, or sidewalks in their neighborhood in 2016). The classification of overweight and obesity is based on body mass index (BMI), with overweight classified between 25 and 30, and 30 or greater classified as obese.

Data on demographic and socioeconomic covariates were obtained from the 2018 American Community Survey (ACS) [32] and the United States Bureau of Labor Statistics. State-level one-year estimates for total population size, the percentage of the population made up of non-Hispanic whites, median household income, unemployment rate, and percent with high school education or greater among those 25 years and older were normalized, and Z-score values were included as covariates in all models.

Analyses included data from all 50 states and the District of Columbia (N = 51). We categorized the state-aggregated built environment indicators into three tertiles (low, medium, and high) such that states with, for instance, the most crosswalks, would be in the third tertile for that indicator. State-level built environment characteristics were merged with health outcome data by state. Health outcomes were modeled as continuous variables (e.g., state-level obesity prevalence). Comparisons of health outcomes across the three tertiles were conducted, using the 1st tertile as the referent group. Adjusted linear regression models were utilized to estimate associations between each state-level built environment indicator and each state health outcome separately. Reported results are interpreted as prevalence differences in health outcomes between the third and second tertile vs. the first tertile. A positive number indicates that the third or second tertile (vs. the first tertile) had a higher prevalence of a health outcome, while a negative number indicates that the third or second tertile had a lower prevalence of a health outcome. Statistical significance in this study was evaluated at α = 0.05. This study was approved on 10 July 2017 by the Institutional Review Board (IRB) at the University of Maryland, College Park (IRB number 1074955-6). Statistical analyses in this study were performed using Stata MP13 (StataCorp LP, College Station, TX, USA).

## 3. Results

### 3.1. Descriptive Characteristics

Descriptive statistics for GSV-derived built environment characteristics and health outcomes are displayed in Table 1. Among the examined built environment indicators, visible wires were the most common, with a mean prevalence of 61.03% among states, while crosswalks were the least common, with a mean prevalence of 6.24% among states. For state-level outcomes, the average prevalence of adult obesity among states was 30.71%, while the average prevalence of overweight adults among states was 35.14%. The average percentages of obese and overweight adolescents among states were lower than the rates for adults, at 14.77% and 15.75%, respectively. The average percentage of adults who engaged in weekly aerobic physical activity among states was almost 50%. In comparison, the average percentage of adolescents engaging in daily physical activity was 23.44% among states.

### 3.2. Associations between Built Environment Indicators and Health Outcomes

#### 3.2.1. Crosswalk Presence (Indicator of Walkability)

More crosswalks were predictive of lower adult and adolescent obesity, as well as decreases in premature mortality (Table 2). Being in the highest tertile for crosswalks was associated with a 2.72% lower prevalence for adult obesity and 81.77 fewer premature deaths per 100,000 (Table 2). States in both the highest and medium tertiles of having crosswalks had adolescent obesity prevalences that were about 2% lower than states with the least crosswalks, although only the medium tertile reached statistical significance (Table 2).

#### 3.2.2. Visible Wire Presence (Indicator of Physical Disorder)

More visible wires were associated with increases in adult and adolescent obesity and decreases in adolescents engaged in aerobic and general physical activity (Table 2). Additionally, more visible wires were associated with increases in cases of premature mortality, diabetes mortality, and cardiovascular mortality (Table 2). However, none of these associations reached statistical significance at the α = 0.05 level.

#### 3.2.3. Building Type: Non-single Family Home (Indicator of Mixed Land Use/Urban Development)

An increased prevalence of non-single family homes was predictive of decreased diabetes prevalence, increased prevalence of adolescents engaging in physical activity, and decreases in cases of premature mortality (Table 2). Increased prevalence in non-single family homes were also predictive of an increase in park access prevalence and youth recreational access prevalence (Table 2). States in the highest tertile for non-single family homes were associated with a 1.40% decrease in diabetes prevalence and 68.82 fewer premature deaths per 100,000 compared to states with the least non-single family homes. States in the highest and middle tertiles of non-single family homes had a prevalence of adolescents engaging in physical activity that were about 3.6% higher than in states with the least non-single family homes (Table 2). Additionally, states in the highest tertile of having non-single family homes were also associated with a 23.20% increase in park access prevalence and an 8.68% increase in youth recreational access compared to states with the least non-single family homes (Table 3).

#### 3.2.4. Single-Lane Road (Indicator of Less Urban Development)

Single-lane roads were used as an indicator of lower urban development. A higher prevalence of single-lane roads was associated with an increased prevalence of adult obesity and a decreased prevalence of park access (Table 2). States in the highest tertile of single-lane roads had a 3.06% increase in adult obesity prevalence and a 13.22% decrease in park access prevalence compared to states in the lowest tertile of having single-lane roads (Table 3).

## 4. Discussion

We set out to examine possible associations between features of the built environment (crosswalks, non-single family home buildings, single-lane roads, and visible wires) and a variety of health behaviors and outcomes, as well as access to facilities that are beneficial to residents’ health and well-being. Our results echoed findings previously published by other investigators, showing associations between indicators of walkability, urban development, and disorder on physical activity, chronic health conditions, and mortality.

A higher frequency of crosswalks in an area indicates improved walkability. We found that more crosswalks were associated with a lower prevalence of adult and adolescent obesity, as well as a decrease in premature mortality. Previous studies have found that infrastructure geared toward increasing walkability increased physical activity and decreased obesity and related morbidities. For example, James and colleagues [3] found that increasing walkability predicted lower BMI among Nurses’ Health Study participants in areas with a high walkability index. Similarly, an ecological analysis of walkability in cities in Southern Ontario found that higher neighborhood walkability was linked to decreases in overweight status, obesity, and diabetes [18]. This indicates that more crosswalks may facilitate better health outcomes, possibly due to increases in physical activity resulting from improvements to neighborhood walkability.

Another finding of the study was that a higher number of non-single family home buildings was associated with decreases in some adverse health outcomes (diabetes and premature mortality). This indicator also predicted increases in healthy behaviors (adolescents engaging in physical activity) and access to parks and recreational sources. This built environment feature serves as an indicator of mixed land use, which is a reliable differentiator between purely residential areas (i.e., suburban areas, which have a higher prevalence of single-family homes) and urban areas with a mixture of houses and other building types. These results, which suggest that more urban development is linked to decreases in chronic health conditions and morbidities, as well as more positive health behaviors such as physical activity, are similar to those from previous analyses we have conducted using GSV to measure urban development [19,20]. In sum, higher levels of urban development could facilitate healthier behaviors which lead to better health outcomes, particularly for youth.

Similarly, we found that an increased frequency of single-lane roads was associated with a higher prevalence of adult obesity, as well as lower park access. As opposed to non-single family home buildings, which indicate higher urbanicity, more single-lane roads may indicate lower levels of urban development, as they are more common in suburban and rural areas. Researchers have found that rural residence is associated with a higher BMI and obesity [21] and that cardiovascular disease is more prevalent among rural populations [22]. Some of these disparities may be explained by decreased access to park facilities and places to exercise. Previous analyses have concluded that both adults and youths in rural areas are less likely to be physically active, putting them at risk of obesity and related morbidities [23].

There are several strengths that characterize this study. Our use of a large number of GSV images (n = 31,247,167) from across the U.S. provides nearly complete coverage of the country. This coverage, combined with the use of deep learning techniques to characterize built environment features, ensures consistent characterization of the built environment over a large, continuous geographic region. The health outcomes we examined covered a diverse range of chronic conditions, health behaviors, and access to neighborhood resources that affect multiple facets of individual well-being. Moreover, our analysis was carried out at the state level in the U.S. While lacking some of the nuance of an analysis using a smaller geographic unit, the conclusions drawn at the state level may be more generalizable and more easily incorporated into policymaking.

Despite its strengths, our analysis is also subject to limitations. Because it is an ecological analysis, the inferences that can be drawn from the results should be regarded with caution. Data on health outcomes including obesity, diabetes, cardiovascular disease, and mortality were collected by CDC in 2014, resulting in the potential for a temporal mismatch between built environment features (as GSV images have been taken continuously since the program’s inception) and measured outcomes. Moreover, there are disparities in the availability of Google Street View images by urbanicity. Google Street View has fewer images in rural areas and updates rural imagery less frequently. In addition, other neighborhood indicators not captured by our computer vision models may also be important determinants of the outcomes examined. For instance, the availability of public transportation, such as bus stops and metro stations, might encourage daily physical activity [33]. We examined park access but not park size or other park characteristics that may impact health outcomes. As previous research has found, neighborhood risks and resources tend to correlate with, for instance, economically disadvantaged neighborhoods having multiple indicators of lower neighborhood quality [34]. In this study, analyses presented associations between each individual built environment indicator and health outcomes. However, this could have inflated the number of statistically significant associations detected. Additionally, while we controlled for sociodemographic characteristics, residual confounding could still be present.

## 5. Conclusions

In our study, select built environment features were significantly associated with multiple chronic health outcomes, health behaviors, and access to recreational facilities. Crosswalks were associated with reductions in obesity and premature mortality. Non-single family homes were associated with decreased diabetes and premature mortality, as well as increased physical activity and park and recreational access. Lastly, single-lane roads were associated with increased obesity and decreased park access. We believe our study will contribute to a filling of the knowledge gap concerning the built environment’s impact on obesity and physical activity by using deep-learning-derived indicators to characterize neighborhood features.

## Figures and Tables

**Table 1:** Descriptive Statistics, state level.

	Mean (SD)
Google Street View	
Crosswalks	6.24% (6.38)
Non-single family home	32.18% (12.89)
Single lane	53.58% (8.66)
Visible wires	61.03% (7.45)
Other built environment characteristics	
Park access	45.35 (19.38)
Youth recreational access	39.34 (12.19)
State health outcomes	
Adult obesity (prevalence)	30.71 (3.82)
Adult overweight (prevalence)	35.14 (1.35)
Adolescent obesity (prevalence)	14.77 (3.12)
Adolescent overweight (prevalence)	15.75 (1.49)
Adult diabetes (prevalence)	9.59 (1.75)
Percentage of adults engaging in weekly aerobic physical activity	49.85 (6.16)
Percentage of adolescents engaging in daily physical activity	23.44 (3.70)
Premature mortality (cases per 100,000)	609.01 (125.76)
Diabetes mortality (cases per 100,000)	67.51 (14.87)
Cardiovascular disease mortality (cases per 100,000)	220.78 (33.25)

Data sources for health outcomes: 2014–2016 CDC CDI for premature mortality, diabetes mortality, cardiovascular mortality, cardiovascular disease mortality; 2015–2017 CDC DNPAO for obesity, overweight, physical activity, park access, and recreational access. SD—standard deviation.

**Table 2:** Associations between state health outcomes and built environment characteristics ^a.^

	% Adult Obesity	% Adolescent Obesity	% Diabetes	% Adults Engaged in Aerobic Physical Activity	% Adolescents Engage in Physical Activity	Premature Mortality	Diabetes Mortality	Cardiovascular Disease Mortality
	Prevalence Difference	Prevalence Difference	Prevalence Difference	Prevalence Difference	Prevalence Difference	Cases per 100,000	Cases per 100,000	Cases per 100,000
	(95% CI)	(95% CI)	(95% CI)	(95% CI)	(95% CI)	(95% CI)	(95% CI)	(95% CI)
Crosswalk								
High	−2.72 * (−5.28, −0.17)	−1.85 (−4.05, 0.35)	−0.75 (−1.58, 0.07)	0.46 (−2.86, 3.78)	−0.17 (−3.32, 2.97)	−81.77 * (−147.67, −15.87)	−6.13 (−18.22, 5.96)	−10.93 (−34.14, 12.27)
Medium	−0.69 (−2.97, 1.59)	−2.21 * (−4.23, −0.19)	−0.65 (−1.38, 0.08)	−1.64 (−4.60, 1.32)	0.77 (−2.11, 3.66)	−66.85 * (−125.68, −8.02)	−9.84 (−20.64, 0.95)	−14.67 (−35.38, 6.05)
Visible wires								
High	0.64 (−1.70, 2.98)	1.37 (−0.67, 3.41)	0.05 (−0.70, 0.79)	−2.48 (−5.34, 0.38)	0.51 (−2.30, 3.32)	19.03 (−42.39, 80.45)	7.52 (−2.77, 17.80)	8.10 (−12.31, 28.51)
Medium	0.19 (−1.82, 2.21)	0.05 (−1.81, 1.91)	0.11 (−0.53, 0.75)	−0.57 (−3.03, 1.89)	0.38 (−2.18, 2.95)	−3.41 (−56.20, 49.38)	−3.96 (−12.80, 4.88)	−0.77 (−18.31, 16.77)
Non-single family home								
High	−2.18 (−4.43, 0.08)	−1.74 (−3.96, 0.48)	−1.40 (−2.04, −0.75)	1.26 (−1.79, 4.31)	3.55 * (0.92, 6.17)	−68.82 (−129.02, −8.61)	−2.09 (−13.33, 9.16)	−17.36 (−38.05, 3.33)
Medium	0.68 (−1.40, 2.77)	−0.41 (−2.36, 1.55)	−0.64 (−1.24, −0.05)	0.34 (−2.49, 3.17)	3.63 * (1.32, 5.95)	−48.19 (−103.97, 7.60)	1.57 (−8.84, 11.99)	−9.53 (−28.70, 9.64)
Single lane roads								
High	3.06 * (0.90, 5.22)	1.46 (−0.60, 3.51)	0.39 (−0.35, 1.13)	−1.72 (−4.66, 1.22)	0.01 (−2.83, 2.84)	48.23 (−10.71, 107.16)	3.68 (−7.16, 14.51)	19.57 (−0.13, 39.27)
Medium	2.11 * (0.16, 4.07)	0.66 (−1.31, 2.64)	0.14 (−0.53, 0.81)	−1.14 (−3.80, 1.52)	0.17 (−2.55, 2.89)	−11.30 (−64.59, 42.00)	−1.80 (−11.59, 8.00)	2.56 (−15.25, 20.38)

^a^—Adjusted linear regression models were run for each outcome separately. Built environment features were categorized into tertiles (high, medium, low). Models controlled for total population size, percent non-Hispanic white, unemployment rate, median household income, and percent with high school education or greater among those 25 years and older; N = 51; *—*p* < 0.05. CI—confidence interval.

**Table 3:** Associations between state-level recreational access and built environment characteristics ^a^.

	% Park Access	% Youth Recreational Access
	Prevalence Difference (95% CI)	Prevalence Difference (95% CI)
*Non-single family home*		
High	23.20 (15.01, 31.38) *	8.68 (2.71, 14.66) *
Medium	18.43 (10.85, 26.01)*	5.38 (−0.16, 10.92)
*Single-lane roads*		
High	−13.22 (−22.99, −3.45) *	−5.36 (−11.48, 0.77)
Medium	−13.17 (−22.01, −4.34) *	−4.41 (−9.95, 1.12)

^a^—Adjusted linear regression models were run for each outcome separately. Built environment features were categorized into tertiles (high, medium, low). Models controlled for total population size, percent non-Hispanic white, unemployment rate, median household income, and percent with high school education or greater among those 25 years and older. *—*p* < 0.05. CI—confidence interval.
